# Supplementary material for: 70 km long-range Raman distributed optical fibre sensing through enhanced anti-distortion coding and waveform reconstruction
Source: Nat Commun. 2025 Dec 15;17:676. doi: 10.1038/s41467-025-67314-2 (PMC12820281; doi:10.1038/s41467-025-67314-2)
Supplement: Supplementary file 1 — Supplementary Information [file 41467_2025_67314_MOESM1_ESM.pdf]

Supplementary Information:

70 km Long-Range Raman Distributed Optical Fibre Sensing Through Enhanced Anti-distortion Coding and Waveform Reconstruction

Fan Zhang [1,2], Jian Li [1,2,3,*], Lulei Li [1,2], Kangyi Cao [1,2], Mingjiang Zhang [1,2,3,*]

[1] College of Physics and Optoelectronics, Taiyuan University of Technology, Taiyuan 030024, China

[2] Key Laboratory of Advanced Transducers and Intelligent Control Systems (Ministry of Education and Shanxi Province), Taiyuan University of Technology, Taiyuan 030024, China

[3] Shanxi Key Laboratory of Precision Measurement Physics, Taiyuan University of Technology, Taiyuan 030032China

*Corresponding author(s). E-mail(s):

lijian02@tyut.edu.cn,zhangmingjiang@tyut.edu.cn

Metrics and limitations of existing coding schemes

Currently, pulse coding schemes applied in Raman distributed optical fiber sensing system are primarily classified into five categories, which specifically include the Simplex coding scheme, low-repetition-rate cyclic pulse coding scheme [27], Pre-Shaped Simplex Coding scheme, Genetic-optimised aperiodic coding scheme [29], Derived Sequences coding scheme.

Simplex coding scheme, as a linear coding technique based on Hadamard matrix transformation, has its code length (in l bits) determining the number of detection signals required (l groups). For instance, in 2006, J. Park, et al. achieved a spatial resolution of 17.0 m, a temperature resolution of 3.0 °C, and a total of 2176 effective sensing points over a sensing distance of 37.0 km by utilizing this coding scheme and link optimization techniques. The main limitation of this scheme lies in the linear growth of data acquisition volume and computational complexity with code length, which restricts its practical efficiency in applications requiring long code lengths and high-speed sensing.

Low-repetition-rate cyclic pulse coding scheme generates Simplex coding-based cyclic coding sequences using an acousto-optic modulator (AOM), enabling pulses to be injected into the optical fiber periodically at a low repetition rate. For example, in 2011, M. A. Soto, et al. combined the low-repetition-rate quasi-periodic cyclic pulse coding with a high-power fiber laser, achieving a spatial resolution of 1.0 m, a temperature resolution of 3.0 °C, and 26,000 effective sensing points over a sensing distance of 26.0 km. However, higher pulse energy also makes it more likely to induce fiber nonlinear effects, and when approaching the stimulated scattering threshold, it restricts the system’s sensing distance and SNR performance.

Pre-Shaped Simplex Coding scheme is based on simplex coding technology and adopts a linearly increasing profile to adjust pulse amplitude, compensating for the amplitude variation caused by EDFA to suppress the transient effect of the EDFA and improve system performance. For example, in 2017, J. B. Rosolem, et al. achieved a spatial resolution of 10.0 m, a temperature resolution of 8.4 °C, and 6,200 effective sensing points over a sensing distance of 62.0 km by using the pre-shaped Simplex coding and a gain-controlled erbium-doped fiber amplifier. Although this scheme effectively suppresses the transient response of the EDFA at the hardware level, its hardware structure is more complex, leading to a significant increase in implementation cost.

Genetic-optimised aperiodic coding scheme utilizes a distributed genetic algorithm (DGA) to generate a single-sequence aperiodic code (GO-code), which is then converted into an optical pulse sequence and injected into the optical fiber. For example, in 2020, X. Z. Sun, et al. achieved a spatial resolution of 1.0 m, a temperature resolution of 3.9 °C, and 39,000 effective sensing points over a sensing distance of 39.0 km based on the Genetic-optimised aperiodic coding scheme. The core challenge of this scheme lies in its high dependence on optimization algorithms to search for the optimal code, which typically requires substantial computational resources and iterative testing.

Derived Sequences coding scheme addresses the transient effects of EDFA through a derived sequence decoding method, thereby enhancing the performance of long-distance sensing. For instance, in 2022, D. D. Chai, et al. achieved a spatial resolution of 5.0 m, a temperature resolution of 2.5 °C, and 8,800 effective sensing points over a sensing distance of 44.0 km based on the Derived Sequences coding scheme. However, this scheme's decoding process imposes a heavy computational burden, and the core algorithm involves high-complexity matrix operations, making it difficult to meet real-time requirements.

However, existing pulse coding techniques in Raman distributed optical fiber sensing remain constrained by several prevalent bottlenecks: high hardware implementation complexity, excessive computational overhead in encoding and decoding, limited nonlinear effect suppression capability, and inadequate resistance to signal distortion coupled with poor stability during long-distance transmission. Collectively, these factors constrain the system's application potential in high-performance scenarios—including low SNR environments and long-distance sensing applications.

Comparison with existing coding schemes

Currently, the pulse coding techniques applied in Raman distributed optical fiber sensing system mainly include the following: Simplex coding scheme, Pre-Shaped Simplex coding scheme, Low-repetition-rate cyclic pulse coding scheme, Genetic-optimised aperiodic coding scheme, and Derived Sequences coding scheme.

Supplementary Table 1. Sensing performance of various coding schemes.

Scheme	Sensing Distance	Spatial Resolution	Sensing Points	Temperature Resolution	Measurement Time
Simplex coding [31]	37.0 km	17.0 m	2,176	3.0 ℃	/
Low-repetition-rate cyclic pulse coding [27]	26.0 km	1.0 m	26,000	3.0 ℃	30 s
Pre-Shaped Simplex Coding [32]	62.0 km	10.0 m	6,200	8.4 ℃	/
Genetic-optimised aperiodic coding [29]	39.0 km	1.0 m	39,000	3.9 ℃	13.6 min
Derived Sequences coding [33]	44.0 km	5.0 m	8,800	2.5 ℃	/
EAC-coding	70.0 km	1.58 m	44,303	5.39 ℃	252 min

Sensing Points refers to the number of independent spatial sampling points in the fibre optic sensing link where the system can achieve effective measurements. The specific calculation method is the ratio of the sensing distance to the spatial resolution.
Temperature resolution is defined as the minimum temperature change that a system can reliably distinguish, and its calculation method is based on the standard deviation under steady-state conditions.

We have compared our proposed scheme with other coding methods such as simplex coding and Genetic-optimised aperiodic coding in terms of coding gain, measurement time, and system complexity. The specific revisions are as follows.

In terms of coding gain, the EAC-coding scheme proposed in this paper exhibits significant advantages in SNR improvement. The core of this scheme lies in the analysis and accurate reconstruction of the waveform of Raman backscattered signals, which effectively compensates for the detection signals distortion caused by the transient effects of the system. This mechanism enables EAC-coding to overcome the performance bottleneck of traditional derived sequences coding schemes induced by signals distortion, achieving a coding gain closer to the theoretical limit. Specifically, existing derived sequence coding scheme construct coding sequences based on Golay complementary sequences and perform correlation decoding using their derived sequences. The upper limit of their theoretical coding gain is $\sqrt{L}/2$ (where L denotes the

length of the coding sequence). However, due to the unavoidable distortion (especially transient distortion) in actual detection signals, the results of their correlation operations deteriorate, leading to a significant gap between the actually achieved coding gain and this theoretical value. In contrast, the EAC-coding scheme, through the aforementioned signals analysis and reconstruction mechanism, significantly suppresses the impact of transient distortion on the detection signals waveform, thereby enabling its coding gain to approach the theoretical upper limit of $\sqrt{L}/2$. Benefiting from this, the proposed system ultimately achieves temperature sensing capability in a long-distance Raman distributed optical fiber sensing system with a sensing distance of 70.0 km.

In terms of measurement time, the core advantage of the EAC-coding scheme stems from its extremely low decoding computational complexity. This scheme achieves signals reconstruction solely through Fourier transform, which significantly reduces the computational cost and time delay of real-time data processing, thereby effectively improving the overall response speed of the system. Specifically, for a Simplex code with length L, it is necessary to inject L groups of detection signals into the optical fiber and collect L groups of Raman backscattered signals, with its theoretical minimum acquisition time being $T_{\text{Simplex}}=L\times t$ (where t denotes the acquisition time of one group of Raman backscattered curves). Although the Genetic-optimised aperiodic coding scheme only requires injecting one group of detection signals into the optical fiber, it needs additional time to design the coding sequence based on the distributed genetic algorithm. The Derived sequences coding scheme requires injecting 4 groups of detection signals into the optical fiber, with a theoretical acquisition time of $4t$. However, in the decoding stage, it involves complex steps such as extracting the attenuation envelope, establishing a compensation model, and solving the compensation envelope via matrix operations, which increases the time cost of the decoding process. The EAC-coding scheme proposed in this paper performs encoding based on Golay complementary sequences, with a theoretical acquisition time of $4t$, and only needs Fourier transform to complete the analysis and reconstruction of the Raman backscattered signals waveform. This significant efficiency improvement at the algorithm level greatly reduces the real-time data processing delay and effectively enhances the system response speed, providing crucial real-time guarantee for the final realization of temperature sensing in a 70.0 km long-distance Raman distributed optical fiber sensing system.

In terms of system complexity, the core innovation of the EAC-coding scheme lies in achieving a high degree of simplification in the decoding process through algorithm

optimization, while keeping the hardware architecture unchanged. This scheme only needs to perform efficient Fourier transform operations to complete signal reconstruction, which significantly reduces the algorithmic complexity and computational burden of real-time data processing. The specific reasons are as follows. Simplex coding scheme requires the injection of L groups of detection signals, resulting in a long acquisition time and a large volume of raw data. This not only imposes high requirements on hardware performance (such as data acquisition rate and storage capacity) and long-term stability, but also the large-scale data processing involved in its decoding process significantly increases the computational complexity. Genetic-optimised aperiodic coding scheme greatly shortens the acquisition time without increasing hardware costs. However, due to its unique design, it is necessary to design coding sequences based on a distributed genetic algorithm and conduct extensive tests to find the optimal coding sequence. Derived sequences coding scheme also does not require additional hardware overhead. However, this scheme introduces high computational complexity in the decoding stage, as it relies on computationally expensive matrix operations to generate and process derived sequences.

EAC-coding scheme proposed in this paper incorporates a novel preprocessing framework for autocorrelation characteristic analysis, waveform analysis and reconstruction of Raman backscattered signals. This framework significantly improves the fidelity and transmission stability of the encoded pulse sequence by dynamically compensating for the transient gain fluctuation of EDFA. This technology leverages the inherent gain of EAC-coding pulse coding to effectively offset fiber scattering attenuation during 70.0 km long-distance transmission, achieving a significant improvement in the baseline SNR. On this basis, a two-stage noise suppression method is further constructed. The first-stage processing involves compensating for signal attenuation using the theoretical coding gain of EAC-coding. For the second-stage processing, a Haar wavelet denoising algorithm is adopted. Its step-matching characteristic enables accurate extraction of the abrupt temperature change features of the sensing fiber and adaptive filtering of the residual time/frequency domain coupled noise in the decoded signal. This three-fold synergistic mechanism, consisting of the preprocessing framework compensating for EDFA fluctuation, coding gain suppressing transmission losses, and wavelet transform finely removing residual noise, breaks through the theoretical trade-off bottleneck between SNR and sensing distance in traditional schemes. Experiment results indicate that, at an ultra-long distance of 70.0 km, this scheme can achieve a spatial resolution of 1.58 m, a temperature resolution of

5.39 °C, and an effective number of sensing points reaching 44,303, verifying the engineering feasibility of the scheme. To the best of our knowledge, the number of effective sensing points achieved by this scheme ranks the highest in the field of Raman distributed optical fiber sensing systems.

The Specific Principles of the EAC-Coding Scheme

The specific principle is shown below. Golay complementary sequences are a class of sequences characterized by superior autocorrelation properties. The unique feature enables precise recovery of single-pulse response, making them widely applicable in information science, particularly in signal processing and communication systems. The autocorrelation function of Golay complementary sequences exhibit a δ function profile. Taking the sequences *A* and *B* as an example, their auto-correlation properties are mathematically expressed by Eq. (1).

$$A * A + B * B = 2L\delta \tag{1}$$

Where * is the correlation operator, and *L* is the sequence code length. The restriction of optical fibers to unipolar pulse propagation prevents the direct use of bipolar Golay complementary sequences (with elements "1" and "-1"), unlike in electrical or acoustic systems, thereby requiring adaptive encoding approaches (modulation with bias) to map bipolar codes onto unipolar optical signals.

$$\begin{cases} u = \dfrac{(1+A)}{2} & w = \dfrac{(1+B)}{2} \\ \bar{u} = \dfrac{(1-A)}{2} & \bar{w} = \dfrac{(1-B)}{2} \end{cases} \tag{2}$$

As shown in Eq. (2), sequences *A* and *B* can bedecomposed into terms involving u, $\bar{u}$, w and $\bar{w}$, yielding the expressions $A=u-\bar{u}$ and $B=w-\bar{w}$. For the pulse $\boldsymbol{u}$ encoded based on Golay complementary sequences, the Raman backscattered signal intensity generated in the system is mathematically expressed through Eq. (3).

$$I = u \otimes h_k \tag{3}$$

Where *I* is the intensity of the Raman backscattered signal; u is the encoded pulse; $\otimes$ is the convolution operator; h_k is the pulse response. The mathematical definition of h_k is explicitly provided in Eq. (4).

$$h_k = K_{as} \cdot S \cdot v_{as}^4 \cdot \phi_e \cdot R_{as}(T) \cdot \exp\left[-(\alpha_0 + \alpha_{as}) \cdot L\right] \tag{4}$$

Supplementary Table 2. Parameters of the Raman anti-Stokes scattering signal

Parameters	Symbol
Raman backscattered coefficients	K_{as}
Raman backscattered factor	S
Frequency of Raman anti-Stokes	v_{as}
Luminous flus	ϕ_e
Loss coefficient	a_0+a_{as}
Length of sensing fiber	L
Temperature modulation function	$R_{as}(T)$

The various parameters of the Raman anti-Stokes scattering signal are specifically specified in supplementary Table 2.

In Raman distributed fiber sensing system, higher incident optical power corresponds to an improved SNR. Consequently, amplification of the incident light is typically implemented using EDFA. However, the transient effects of EDFA can significantly degrade sensing performance. This phenomenon induces non-uniform amplification, leading to waveform distortion of the encoded pulse after passing through the EDFA. Temporally, this manifests as a gradual attenuation profile, which disrupts the autocorrelation properties of the encoded pulse. Consequently, the system experiences both a degraded SNR and a constrained maximum sensing distance. The functional representation of this distortion mechanism is formally defined in Eq. (5).

$$u(f) = u \cdot f \tag{5}$$

Where u(f) is the pulse amplified by EDFA, the attenuation envelope *f* is induced by transient effects. The transient effects inherent to EDFA critically distort the waveform of encoded signals, compromising their integrity in correlation-based sensing systems. The intensity of the Raman backscattered signal generated by the signal *u(f)* is governed by the relationship expressed in Eq. (6).

$$I_u = (u \cdot f) \otimes h_k \tag{6}$$

As demonstrated by Eq. (6), all four segments of the acquired backscattered signals are subjected to transient effects, resulting in attenuation of the backscattered intensity. To mitigate transient effects, a preprocessing scheme is implemented during the decoding stage for Raman backscattered signals. This involves analyzing the distorted signals and reconstructing their waveforms, thereby restoring the autocorrelation properties compromised by transient effects. Taking the encoded pulse u as an example, the attenuation envelope induced by EDFA transient effects is reformulated from a multiplicative operation to a convolutional operation, the Raman backscattered signal is mathematically formulated as shown in the following equation:

$$I_u = u \otimes f' \otimes h_k \tag{7}$$

Where *f'* is the transient-modulated function. The function *f'* does not require explicit analytical definition, as its functional form is fully determined by the attenuation envelope *f*, as formalized in the following equation:

$$f' = \text{ifft}\left[\frac{\text{fft}(u \cdot f)}{U}\right] \tag{8}$$

Where fft is the fast Fourier transform operation; ifft is inverse fast Fourier transform operation; U is the Fourier transform of the encoded pulse u. The Raman backscattered signal in Eq. (9) is subjected to Fourier transform processing, as mathematically expressed in the following equation:

$$\text{fft}(I_u) = U \cdot F' \cdot H_k \tag{9}$$

Where the Fourier transforms of the encoded pulse *u*, the transient-modulated function *f'* and pulse response h_k are represented by *U*, *F'* and H_k. The reconstructed Raman backscattered signal through inverse Fourier transform processing is mathematically formulated in Eq. (10).

$$I'_u = \text{ifft}\left(\frac{\text{fft}(I_u)}{F'}\right) = u \otimes h_k \tag{10}$$

Comparison of Eq. (10) with Eq. (3) reveals that, the transient effects in the Raman backscattered signal are fully eliminated after waveform-reconstruction. The autocorrelation properties can be recovered by implementing the same preprocessing protocol on the remaining three Raman backscattered signals. And decoding is performed on the four waveform-reconstructed Raman backscattered signal.

$$\begin{cases} I_A = A * \left(u \otimes h_k - \bar{u} \otimes h_k\right) \\ I_B = B * \left(w \otimes h_k - \bar{w} \otimes h_k\right) \end{cases} \tag{11}$$

Here, I_A and I_B represent the correlation results between the decoded backscattered signals and the encoding sequences, where * is the correlation operation. Summing I_A and I_B yields Eq. (12).

$$I = I_A + I_B = 2l \cdot \delta_k \otimes h_k \tag{12}$$

Here, δ_k is the single-pulse, and *l* is the code length of the EAC-coding scheme. Consequently, the correlation result is equivalent to the Raman backscattered signal of the single-pulse, thereby reconstructing the backscattered signal of equivalent single-pulse as:

$$\begin{aligned} I(T) &= P \cdot K_{as} \cdot S \cdot v_{as}^4 \cdot R_{as}(T) \cdot \exp\left[-\left(\alpha_0 + \alpha_{as}\right)L\right] \\ &= \frac{I_A + I_B}{2l} \end{aligned} \tag{13}$$

Where P is the incident pulse intensity. The intensity of the Raman backscattered signal under room temperature conditions (T_0) is governed by Eq. (14).

$$I(T_0) = P \cdot K_{as} \cdot S \cdot v_{as}^4 \cdot R_{as}(T_0) \cdot \exp\left[-\left(\alpha_0 + \alpha_{as}\right)L\right] \tag{14}$$

The temperature of the fiber under test (FUT) can be demodulated by applying Eq. (13) and Eq. (14).

$$T = -\frac{h\Delta v}{k \cdot \ln\left\{1 + \frac{I(T_0, L)}{I(T, L)} \cdot \left[\exp\left(-\frac{h\Delta v}{kT_0}\right) - 1\right]\right\}} \tag{15}$$

Following the optical time-domain reflectometry (OTDR) principle, the FUT position is resolved through:

$$L = \frac{ct}{2n} \tag{16}$$

Where c is the speed of light in a vacuum, t is the time required for the backscattered signal generated at position L of the sensing fiber to return to the launch end, and n is the refractive index of the fiber.

The specific experimental setup

We built a Raman distributed optical fiber sensing experimental setup based on proposed EAC-coding scheme, as shown in Supplementary Fig. 1. Firstly, the laser generates continuous light with a central wavelength of 1550 nm, and then the detection signals encoded based on Golay complementary sequences are generated by using the digital delayed pulse generator (DDPG) to modulate the semiconductor optical amplifier (SOA). Each detection laser signal is amplified by EDFA and then enters a single-mode sensing fiber through a wavelength division multiplexer (WDM), and generates Raman backscattered signal (RBS) which enters an avalanche photodetector (APD) for amplification and opto-electronic conversion. Finally, all Raman backscattered signals generated by detection signals are collected by a data acquisition card (DAC) and demodulated in a special computer to obtain temperature information.

The equipment used in the experiment, along with their corresponding model numbers, manufacturers, and key optical parameters, are listed as follows: Laser (KEYANG PHOTONICS; DFB Continuous laser; Center Wavelength:1550 nm);SOA (OPEAK; OAM-SOA-PL-15-15-S; Operating Wavelength: 1520-1570 nm); DDPG (CIQTEK; ASG8100; Maximum Coding bit: 99 bit); EDFA (OPEAK; EDFA-C-PL-MB-100-S; Operating Wavelength: 1550 nm); WDM (OPEAK; WDM-1*3-1550; Isolation: ≥60 dB); APD (KEYANG PHOTONICS; KY-DTS-200M; Bandwidth:200 MHz); DAC (CIQTEK; DAQ2100; Sampling Rate: 1 GSa/s). And all experimental equipment used in this experiment are conventional commercial products available on the market.

In Raman distributed optical fiber sensing system, the transient effects of EDFA significantly impacts sensing performance. When the intensity of laser input to the EDFA changes rapidly, the energy level distribution of erbium ions in the EDFA cannot immediately keep up with the change in the intensity of input laser, causing transient gain fluctuation. This phenomenon will lead to a non-uniform amplification that the detection signals will distort in timing when passing through the EDFA, thereby disrupting the autocorrelation of the detection signals, and ultimately decreasing SNR of system while limiting maximum sensing distance.

In the proposed EAC-coding scheme experiment, the 64-bit coding sequence employs a 10 ns pulse width per code element. To mitigate interference between encoded signals and forward Raman scattering components, a 33.3% duty cycle return-to-zero format is implemented. The sensing fiber is single-mode fiber with a length of 70.0 km to avoid the deterioration of spatial resolution caused by intermodal dispersion. Each Raman backscattered signal is acquired for 63 min and averaged over 3 million times. Furthermore, the measurement time of the scheme proposed in this paper is mainly limited by the massive data volume (with the effective number of sensing points reaching 44,303) and the hardware accumulation processing time of the acquisition card, which are caused by its long sensing distance and high spatial resolution—rather than

the complexity of its processing architecture. The current measurement time (252 min) is constrained by the processing capability of the data acquisition card (acquisition rate: 1.0 GSa/s; storage depth: 512 Mpts). The massive data volume (approximately 700 k points per frame corresponding to a 70.0 km sensing distance) exceeds the on-board real-time processing limit. In the future, high-performance acquisition cards will be upgraded to optimize the data processing pipeline, thereby eliminating the delay deterioration caused by hardware bottlenecks.

In the experimental process of this scheme, only the Raman anti-Stokes scattering signals were analyzed and processed, and the subsequent temperature demodulation was also performed solely based on the Raman anti-Stokes scattering signals. Compared with the dual-path demodulation scheme (where Raman Stokes scattering light is used to demodulate Raman anti-Stokes scattering signals), this scheme has the following two specific advantages. The Raman anti-Stokes scattering signal based on single-path demodulation exhibits higher sensitivity to the temperature distribution along the sensing fiber. The single-path demodulation scheme can enhance the system's measurement rate and reduce system costs.

To ensure the environmental temperature stability of the sensing fiber during the long-term experiment, the entire 70.0 km sensing fiber is placed in an ultra-clean constant-temperature laboratory, where the ambient temperature is maintained at 24.0±1.0 °C. The FUT is placed in a high-precision constant-temperature water bath with a temperature fluctuation of less than 0.10 °C. Thus, the above experimental setup can guarantee the temperature stability of the testing environment for the sensing fiber during long-term measurements.
